# High capacity data hiding scheme based on (7, 4) Hamming code

**DOI:** 10.1186/s40064-016-1818-0

**Published:** 2016-02-25

**Authors:** Zekun Cao, Zhaoxia Yin, Honghe Hu, Xiangping Gao, Liangmin Wang

**Affiliations:** Key Laboratory of Intelligent Computing & Signal Processing, School of Computer Science & Technology, Anhui University, Hefei, 230601 Anhui China; School of Communication and Information Engineering, Shanghai University, Shanghai, 200072 P.R. China

**Keywords:** Data hiding, Hamming code, Image quality, Embedding capacity

## Abstract

Aiming to embed large amount of data while minimize the sum of costs of all changed pixels, a novel high capacity data hiding scheme based on (7, 4) Hamming code is realized by a family of algorithms. Firstly, *n* (*n* = 1, 2, 3) cover pixels are assigned to one set according to the payload. Then, 128 binary strings of length seven are divided into eight sets according to the syndrome of every binary string. Binary strings that share the same syndrome are classified into one set. Finally, a binary string in a certain set determined by the data to be embedded is chosen to modify some of the least significant bits of the *n* cover pixels. The experimental results demonstrate that the image quality of the proposed method with high embedding payload is superior to those of the related schemes.

## Background

Data hiding, frequently interchangeably referred to as information hiding, is the art of embedding additional data in a certain carrier (Zielińska et al. 2014). These carriers are typically digital media files transmitted on the Internet, such as images, audios, videos, or text (Ker et al. 2013). Historically, the design of data hiding schemes for digital images has heavily relied on heuristic principles (Feng et al. 2015; Hong et al. 2015; Qian and Zhang 2015; Xia et al. 2014a, b). The current trend calls for constraining the embedding changes to image segments with complex content. Such adaptive data hiding schemes are typically realized by first defining the cost of changing each pixel and then embedding the additional data while minimizing the sum of costs of all changed pixels. One of the explorations to achieve this goal is applying the error correcting code to data hiding, and many researchers have done a lot of research in this area (Chang and Chou 2008; Chen et al. 2013; Liu 2007; Ma et al. 2013; Wang 2009; Yin et al. 2010; Zhang et al. 2007; Zhu et al. 2010).

Crandall originally proposed a data hiding scheme named matrix encoding (Crandall 1998) in 1998. In this scheme, *k* bits were embedded into 2^*k*^ − 1 cover pixels by modifying the least significant bits (LSBs) of one pixel. The embedding capacity reached *k*/(2^*k*^ − 1) bit per pixel (bpp). Based on the matrix encoding, Zhang et al. (2007) proposed the “Hamming+1” scheme in 2007. Compared with the matrix encoding scheme, it used one more cover pixel to embed one more bit while the cost remain unchanged. Thus, the embedding capacity got increased to be (*k* + 1)/2^*k*^ bpp. Later, Chang et al. proposed a new scheme (Chang and Chou 2008) based on the idea of classification in 2008. Binary strings were assigned into eight sets. A binary string of length 2^*k*^ − 1 in a specific set was selected out to embed *k* bits. It presented a new idea in applying Hamming code to data hiding. But the embedding capacity didn’t get improved compared with the previous two scheme. It is equal to the embedding capacity of the matrix encoding scheme (Crandall 1998).

The marked-image quality of the aforementioned schemes is ideal when the embedding payload is low (no more than *k*/(2^*k*^ − 1) or (*k* + 1)/2^*k*^ bpp), but it degrades hardly with the increase of the embedding payload. Against this problem, a new data hiding scheme based on (7, 4) Hamming code is proposed in this paper. The marked-image quality of the proposed scheme is superior to those of the related works in Crandall (1998), Zhang et al. (2007) and Chang and Chou (2008) under a high embedding payload.

## Related works

### The Hamming code

An error correcting code could not only detect that errors have occurred but also locate the error positions. Hamming code is a linear error correcting code that can detect and correct single bit errors. The (*n*, *n* − *k*) Hamming code uses *n* cover bits to transmit *n* − *k* message bits, and the other *k* bits used for error correcting purpose are called parity check bits, where *n* = 2^*k*^ − 1 on the binary filed.

*S* = {*C*_1_, *C*_2_, …,*C*_*M*_} is a set of code words. The number of elements of *S*, denoted as |*S*|, is called the cardinality of the code. For any two code words *x* = (*x*_1_, *x*_2_, …, *x*_*n*_) ∊ *S* and *y* = (*y*_1_, *y*_2_, …, *y*_*n*_) ∊ *S*, the Hamming distance is defined by *d*_*H*_(*x*, *y*) = |{*i*|*x*_*i*_ ≠ *y*_*i*_}|. The minimum distance of the code *S* is defined as *d*_min_ = min {*d*_*H*_(*x*, *y*)|*x*, *y* ∊ *S*}. And the covering radius of the code *S* is *r* if any binary string *u* = (*u*_1_, *u*_2_, …, *u*_*n*_) differs from at least one code word *x* = (*x*_1_, *x*_2_, …, *x*_*n*_) ∊ *S* in at most *r* positions. The minimum distance *d*_min_ measures the error-correcting capability, and the maximum distortion that occurs when a binary string is replaced by a proper code word is measured by the covering radius *r*. Therefore, a large value of the minimum distance *d*_min_ is preferable to the purpose of error correction whereas a small value of the covering radius *r* is preferable to the purpose of steganography. The (7, 4) Hamming code is a binary code of length *n* = 7, with cardinality |*S*| = 16, minimum distance *d*_min_ = 3, and covering radius *r* = 1.

The (7, 4) Hamming code is now taken as an example to demonstrate how Hamming code correct an error bit. Suppose that the message bits are *m* = (1010). First, the code generator matrix ***G*** is used to form *n* cover bits *C* as follows.

$$C = m \times {\varvec{G}} = (1010) \times \left[ {\begin{array}{*{20}c} 1 & 0 & 0 & 0 & 1 & 1 & 1 \\ 0 & 1 & 0 & 0 & 1 & 1 & 0 \\ 0 & 0 & 1 & 0 & 1 & 0 & 1 \\ 0 & 0 & 0 & 1 & 0 & 1 & 1 \\ \end{array} } \right] = (1010010)$$

Next, the code word *C* is transmitted to a receiver via a noise communication channel. Supposed that the received code word is *C*′ = (1011010). Then the parity check matrix ***H*** is used to compute the syndrome vector ${\varvec{z}} = (z_{1} ,z_{2} ,z_{3} )$ for checking an error as follows.

$${\varvec{z}} = (H \times C^{{{\prime }{\text{T}}}} )^{\text{T}} = \left( {\left[ {\begin{array}{*{20}c} 1 & 1 & 1 & 0 & 1 & 0 & 0 \\ 1 & 1 & 0 & 1 & 0 & 1 & 0 \\ 1 & 0 & 1 & 1 & 0 & 0 & 1 \\ \end{array} } \right] \times (1011010)^{\text{T}} } \right)^{\text{T}} = (011)$$

The vector ${\varvec{z}}^{\text{T}} = (011)^{\text{T}}$ is identical to the fourth column of the parity check matrix ***H***. Thus, an error is detected at the fourth position of *C*′, and *C*′ is corrected by *C*′ = *C*′ ⊕ *e*_4_ = (1010010), where ⊕ is the exclusive-or operation, and *e*_*i*_, the error pattern, is a unit vector of length *n* with a “1” located at the *i*-th position. If the syndrome vector is ${\varvec{z}} = (000)$, the receiver can conclude that no error has occurred.

### “Matrix Encoding”

In the matrix encoding scheme, a string of *k* bits ${\mathbf{s}} = (s_{1} ,s_{2} , \ldots ,s_{k} )$ is embedded into a group of *n* cover pixels by adding or subtracting one to or from at most one cover pixel, where *n* = 2^*k*^ − 1. Firstly, the syndrome vector ${\varvec{z}} = (z_{1} ,z_{2} , \ldots ,z_{k} )$ is calculated by ${\varvec{z}} = ({\mathbf{c}} \times {\varvec{H}}^{\text{T}} ) \oplus {\mathbf{s}}$, with ${\mathbf{c}} = ({\text{LSB}}(p_{1} ),{\text{LSB}}(p_{2} ), \ldots ,{\text{LSB}}(p{}_{n}))$ and LSB (*p*_*i*_) means the least significant bit of *i*-th pixel *p*_*i*_. ***H*** is the parity check matrix of the (*n*, *n* − *k*) Hamming code. ${\text{T}}$ is the transpose operation, and ⊕ is the exclusive-or operation. Next, if the computed syndrome vector ${\varvec{z}}$ is (0, 0,…, 0), then the group of *n* marked pixels ${\varvec{R}}$ is set to be equal to ${\mathbf{c}}$; otherwise, find the *i*-th column of ${\varvec{H}}$ that is equal to the transposed syndrome vector ${\varvec{z}}^{\text{T}}$. The group of *n* marked pixels ${\varvec{R}}$ is calculated by ${\varvec{R}} = {\mathbf{e}}_{i} \oplus {\mathbf{c}}$, where ${\mathbf{e}}_{i}$ is a unit vector of length *n* with “1” located at the *i*-th position. At the receiving side, a receiver can extract the original binary string ${\mathbf{s}}$ from the received group ${\varvec{R}}$ by ${\mathbf{s}} = {\varvec{R}} \times {\varvec{H}}^{\text{T}}$.

### “Hamming+1”

Zhang et al. proposed the “Hamming+1” scheme (Zhang et al. 2007) to embed a string of (*k* + 1) secret bits ${\mathbf{s}} = (s_{1} ,s_{2} , \ldots ,s_{k + 1} )$ into a group of (*n* + 1)*ψ* cover pixels ${\varvec{p}} = (p_{1} ,p_{2} , \ldots ,p_{n + 1} )$, where *n* = 2^*k*^ − 1, by modifying at most one cover pixel as follows.

1 $$(s_{ 1} ,s_{ 2} , \ldots ,s_{k} ) = ({\text{LSB}}(p_{1} ),{\text{LSB}}(p_{2} ), \ldots {\text{LSB}}(p_{n} )) \times {\varvec{H}}^{\text{T}} )$$

2 $$s_{k + 1} = \left( {\left\lfloor {\frac{{p_{1} }}{2}} \right\rfloor + \left\lfloor {\frac{{p_{2} }}{2}} \right\rfloor + \cdots + \left\lfloor {\frac{{p_{n} }}{2}} \right\rfloor + p_{n + 1} } \right)\bmod 2$$

where ${\varvec{H}}$ is the parity check matrix of the (*n, n* − *k*) Hamming code, T is the transpose operation. This means that the first *kψ* secret bits of ${\mathbf{s}}$ are embedded into the first *n* bits of ${\varvec{p}}$ by using matrix encoding, and the last secret bit of ${\mathbf{s}}$ is embedded by using the function of *nψ* cover pixels ${\varvec{p}}$. The embedding rules proposed in (Zhang et al. 2007) are as follows. If Eq. (1) does not hold, then ${\varvec{p}}_{n + 1}$ is kept unchanged, and one cover pixel ${\varvec{p}}_{i}$(1 ≤ *i* ≤ *n*) needs to be increased or decreased by one to make Eqs. (1) and (2) hold simultaneously. If (1) holds and (2) does not, the first *n* pixels are kept unchanged and last cover pixel ${\varvec{p}}_{n + 1}$ is randomly increased or decreased by one.

At the receiving side, a receiver can extract the first *kψ* secret bits of ${\mathbf{s}}$ by applying the extracting way of the matrix encoding scheme and the last secret bit of ${\mathbf{s}}$ can be extracted by using Eq. (2).

### “Nearest Code”

In the nearest covering code scheme (Chang and Chou 2008), all possible combinations of seven bits are classified into eight sets *G*_0_, *G*_1_, …*G*_7_. There are 16 elements ${\varvec{G}}_{u}^{0} ,{\varvec{G}}_{u}^{1} , \ldots G_{u}^{15}$ in each set *G*_*u*_, where 0 ≤ *u* ≤ 7. And ${\varvec{G}}_{u}^{v}$ satisfies equation $u = {\varvec{G}}_{u}^{v} \times {\varvec{H}}^{\text{T}}$, where 0 ≤ *v* ≤ 15, ${\varvec{H}}$ is the parity check matrix of the (7, 4) Hamming code, T is the transpose operation. A covering code ${\varvec{G}}_{s}^{v}$ with nearest Hamming distance to ${\varvec{P}} = ({\text{LSB}}(p_{1} ),{\text{LSB}}(p_{2} ), \ldots ,{\text{LSB}}(p_{7} ))$ is selected in ${\varvec{G}}_{s}$ according to secret bits ${\mathbf{s}} = (s_{1} ,s_{2} ,s_{3} )$, where the subscript of ${\varvec{G}}_{s}$ is equal to the corresponding decimal number of ${\mathbf{s}} = (s_{1} ,s_{2} ,s_{3} )$. Then, the cover pixels are modified by ${\varvec{G}}_{s}^{v}$. At the receiving side, a legal receiver can extract the original secret bits ${\mathbf{s}}$ from the received group of 7 pixels ${\varvec{R}}$ by ${\mathbf{s}} = {\varvec{R}} \times {\varvec{H}}^{\text{T}}$.

## The proposed scheme

In the proposed scheme, a secret binary string of length three is in a mapping relationship with the error pattern of the (7, 4) Hamming code and then can be embedded into a group of cover pixels. The number of the cover pixels in different groups varies under different embedding payload.

### The preparations

*I* is the cover image sized *H* × *W*, and *marked_I* is the marked-image with data *D* = {*d*_1_, …, *d*_*L*_}embedded, where *d*_*i*_ ∊ {0, 1}, 1 ≤ *i* ≤ *L*. $${\varvec{H}} = \left[ {\begin{array}{*{20}c} 1 & 1 & 1 & 0 & 1 & 0 & 0 \\ 1 & 1 & 0 & 1 & 0 & 1 & 0 \\ 1 & 0 & 1 & 1 & 0 & 0 & 1 \\ \end{array} } \right]$$ is a parity check matrix of the (7, 4) Hamming code. A string of binary bits (*b*_1_*b*_2_…*b*_7_) is the cover of a string of three bits (*d*_*i*_*d*_*i*+1_*d*_*i*+2_), and $(b_{1}^{\prime } b_{2}^{\prime } \ldots b_{7}^{\prime } )$ is the marked-string of (*b*_1_*b*_2_…*b*_7_). *p*_*i*_ is the *i*-th pixel in cover image, and *p*_*i*_′ is the *i*-th pixel in marked-image. *p*_*i*_^*j*^ represents the *j*-th least significant bit of pixel *p*_*i*_. ER, i.e. embedding rate, is calculated as follows.

3 $${\text{ER}} = \frac{L}{H \times W}{\text{bpp}}$$

*N*_*n*_ is the number of groups that *n* (*n* = 1, 2, 3) cover pixels are used to embed a three bits string. And *N*_*n*_ satisfies Formula (4). The first equation of Formula (4) indicates that the number of bits to be embedded is equal to the amount of bits the cover image could bear under a particular embedding rate. And the second equation in Formula (4) requires that the cover pixels we need are less or equal to the pixels the cover image could provide.

4 $$\left\{ \begin{array}{l} 3(N_{1} + N_{2} + N_{3} ) = H \times W \times ER \hfill \\ N_{1} + 2N_{2} + 3N_{3} \le H \times W \hfill \\ \end{array} \right.$$

To modify the cover pixels as less as possible, Formula (4) is processed to obtain Formula (5) based on the following considerations. The top priority scheme, grouping three cover pixels together to embed a three bits string, satisfies Formula (4) when 0 ≤ *ER* ≤ 1. When 1 < *ER* ≤ 1.5, grouping two cover pixels to embed a binary string satisfies Formula (4), but there will be some pixels in the cover image unused. Instead, we embed some secret binary strings into groups of three cover pixels and the others into groups of two cover pixels. Obviously, this scheme causes less modification to cover image than the scheme that only using two cover pixels to embed binary strings. Likewise, we embed some binary strings into groups of two cover pixels and the others into groups of one cover pixel when 1.5 < *ER* < 3. Therefore, adaptive *N*_*n*_ is calculated by Formula (5), which contributes to minimize the sum of costs of all changed pixels.

5 $$\left\{ {\begin{array}{ll} {N_{1} = 0,N_{2} = 0,N_{3} = \frac{L}{3}} &\quad {\left( {0 < {\text{ER}} \le 1} \right)} \\ {N_{1} = 0,N_{2} = L - H \times W,N_{3} = H \times W - \frac{2L}{3}} &\quad {\left( {1 < {\text{ER}} \le 1.5} \right)} \\ {N_{1} = \frac{2L}{3} - H \times W,N_{2} = H \times W - \frac{L}{3},N_{3} = 0} &\quad {\left( {1.5 < {\text{ER}} \le 3} \right)} \\ \end{array} } \right.$$

### The data embedding phase

All binary strings of length seven are classified into eight sets *G*_0_, *G*_1_, …*G*_7_. There are 16 elements in every set ${\varvec{G}}_{u} = \{ {\varvec{G}}_{u}^{v} \}_{v = 0}^{15}$, and ${\varvec{G}}_{u}^{v} = (c_{1} ,c_{2} , \ldots ,c_{7} )$ satisfies equation $u = {\varvec{G}}_{u}^{v} \times {\varvec{H}}^{\text{T}}$, where 0 ≤ *u* ≤ 7. Specific embedding algorithms are as follows.

**Algorithm 1: Internal embedding algorithm**

**Input:**$\left( {b_{1} b_{2} \ldots b_{7} } \right)$, a string of 3 data bits $\left( {d_{i} d_{i + 1} d_{i + 2} } \right)$

**Output:**$$\left( {b_{1}^{{\prime }} b_{2}^{{\prime }} \ldots b_{7}^{{\prime }} } \right)$$

**Step 1:** Find a ${\varvec{G}}_{u}^{v}$ which satisfies $(c_{ 1} c_{ 2} c_{ 3} c_{ 4} ) = (b_{ 1} b_{ 2} b_{ 3} b_{4} )$ in ${\varvec{G}}_{u}$, where $u = \left( {d_{i} d_{i + 1} d_{i + 2} } \right)$;

**Step 2:**$$\left( {b_{1}^{\prime } b_{2}^{\prime } \ldots b_{7}^{\prime } } \right) = {\varvec{G}}_{u}^{v}$$.

**Algorithm 2: External embedding algorithm**

**Input:***I, D*

**Output:***marked_I*

**Step 1:** Calculate *ER* according to Eq. (3) and *N*_1_, *N*_2_, *N*_3_ by Formula (5);

**Step 2:**$(b_{1} b_{2} \ldots b_{7} ) = (p_{i}^{7} p_{i}^{6} \ldots p_{i}^{1} )$, then call Algorithm 1 to get $(b_{1}^{{\prime }} b_{2}^{{\prime }} \ldots b_{7}^{{\prime }} )$, and $p_{i}^{{\prime }} = (p_{i}^{8} b_{1}^{{\prime }} b_{2}^{{\prime }} \ldots b_{7}^{{\prime }} )$, $i \in \{ 1,2,3, \ldots ,N_{1} \}$;

**Step 3:**$(b_{1} b_{2} \ldots b_{7} ) = (p_{i}^{4} p_{i}^{3} p_{i + 1}^{3} p_{i}^{2} p_{i + 1}^{2} p_{i}^{1} p_{i + 1}^{1} )$, then call Algorithm 1 to get $(b_{1}^{{\prime }} b_{2}^{{\prime }} \ldots b_{7}^{{\prime }} )$, and $p_{i}^{{\prime }} = (p_{i}^{8} p_{i}^{7} p_{i}^{6} p_{i}^{5} b_{1}^{{\prime }} b_{2}^{{\prime }} b_{4}^{{\prime }} b_{6}^{{\prime }} )$, $p_{i + 1}^{{\prime }} = (p_{i + 1}^{8} p_{i + 1}^{7} p_{i + 1}^{6} p_{i + 1}^{5} p_{i + 1}^{4} b_{3}^{{\prime }} b_{5}^{{\prime }} b_{7}^{{\prime }} )$, $i \in \{ N_{1} + 1,N_{1} + 3,N_{1} + 5, \ldots ,N_{1} + 2N_{2} - 1\}$;

**Step 4:**$(b_{1} b_{2} \ldots b_{7} ) = (p_{i}^{3} p_{i}^{2} p_{i + 1}^{2} p_{i + 2}^{2} p_{i}^{1} p_{i + 1}^{1} p_{i + 2}^{1} )$, then call Algorithm 1 to get $(b_{1}^{{\prime }} b_{2}^{{\prime }} \ldots b_{7}^{{\prime }} )$, and $p_{i} ' = (p_{i}^{8} p_{i}^{7} p_{i}^{6} p_{i}^{5} p_{i}^{4} b_{1} 'b_{2} 'b_{5} ')$, $p_{i + 1} ' = (p_{i + 1}^{8} p_{i + 1}^{7} p_{i + 1}^{6} p_{i + 1}^{5} p_{i + 1}^{4} p_{i + 1}^{3} b_{3} 'b_{6} ')$, $p_{i + 2} ' = (p_{i + 2}^{8} p_{i + 2}^{7} p_{i + 2}^{6} p_{i + 2}^{5} p_{i + 2}^{4} p_{i + 2}^{3} b_{4} 'b_{7} ')$, $i \in \{ N_{1} + 2N_{2} + 1,N_{1} + 2N_{2} + 4, \ldots ,N_{1} + 2N_{2} + 3N_{3} - 2\}$.

### Example: data embedding

**Fig. 1:**
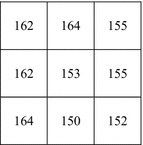
Cover image *I*

An example is now given to demonstrate the embedding phase of the proposed scheme. Suppose that *I* is a grayscale image with *H* × *W* = 3 × 3 shown in Fig. 1 and ${\text{D}} = \{ d_{1} ,d_{2} ,d_{3} , \ldots ,d_{10} ,d_{11} ,d_{12} , \ldots ,d{}_{18}\} = \left\{ {1,0,1,0,0,1,0,0,0,0,1,0,1,0,0,1,1,1} \right\}$.

**Step 1:** We could calculate ER = 2, then work out *N*_1_ = 3, *N*_2_ = 3, *N*_3_ = 0 by Formula (5).

**Step 2:** From the cover image, we know *p*_1_ = 162, $(b_{1} b_{2} \ldots b_{7} ) = (p_{1}^{7} p_{1}^{6} \ldots p_{1}^{1} ) = \left( {0100010} \right),\left( {d_{1} d_{2} d_{3} } \right) = \left( {101} \right)$. Then call Algorithm 1 to get $(b_{1}^{{\prime }} b_{2}^{{\prime }} \ldots b_{7}^{{\prime }} ) = {\varvec{G}}_{5}^{v} = \left( {0100011} \right)$, so $p_{1}^{\prime } = (p_{i}^{8} b_{1}^{\prime } b_{2}^{\prime } \ldots b_{7}^{\prime } ) = 163$. Repeat Step 2 (*N*_1_ − 1) times to embed (*d*_4_*d*_5_*d*_6_) into *p*_2_ = 164 and (*d*_7_*d*_8_*d*_9_) into *p*_3_ = 165.

**Fig. 2:**
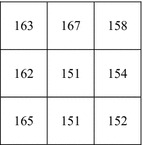
Marked-image *marked_I*

**Step 3:**$\left( {p_{4} ,p_{5} } \right) = \left( {162,153} \right)$, $(b_{1} b_{2} \ldots b_{7} ) = \left( {p_{4}^{4} p_{4}^{3} p_{5}^{3} p_{4}^{2} p_{5}^{2} p_{4}^{1} p_{5}^{1} } \right) = \left( {0001101} \right)$, $\left( {d_{10} d_{11} d_{12} } \right) = \left( {010} \right)$. Call Algorithm 2 to get $(b_{1}^{\prime } b_{2}^{\prime } \ldots b_{7}^{\prime } ) = {\varvec{G}}_{2}^{v} = \left( {0001001} \right)$, so $p_{4}^{\prime } = \left( {p_{4}^{8} p_{4}^{7} p_{4}^{6} p_{4}^{5} b_{1}^{\prime } b_{2}^{\prime } b_{4}^{\prime } b_{6}^{\prime } } \right) = 162$, $p_{5}^{{\prime }} = (p_{5}^{8} p_{5}^{7} p_{5}^{6} p{}_{5}^{5} p_{5}^{4} b_{3}^{{\prime }} b_{5}^{{\prime }} b_{7}^{{\prime }} ) = 151$. Repeat Step 3 (*N*_2_ − 1) times to embed (*d*_13_*d*_14_*d*_15_) into (*p*_6_, *p*_7_) = (155, 164) and (*d*_16_*d*_17_*d*_18_) into (*p*_8_, *p*_9_) = (150, 152). Finally, we get the marked-image *marked_I* shown in Fig. 2.

### The data extracting phase

**Algorithm 3: Data extracting algorithm:**

**Input:***marked_I*, ER

**Output:***D*

**Step 1:** Calculate the value of $N_{n} \left( {n = 1, \, 2, \, 3} \right)$ by Formula (5) according to ER.

**Step 2:**$$(b_{1}^{\prime } b_{2}^{\prime } \ldots b_{7}^{\prime } ) = \left( {p_{i}^{4\prime } p_{i}^{3\prime } p_{i + 1}^{3\prime } p_{i}^{2\prime } p_{i + 1}^{2\prime } p_{i}^{1\prime } p_{i + 1}^{1\prime } } \right)$$, $$\left( {d_{i} d_{i + 1} d_{i + 2} } \right) = (b_{1}^{\prime } b_{2}^{\prime } \ldots b_{7}^{\prime } ) \times {\varvec{H}}^{\text{T}}$$, $$i \in \{ 1, 2, \ldots , N_{1} \}$$.

**Step 3:**$$(b_{1}^{\prime } b_{2}^{\prime } \ldots b_{7}^{\prime } ) = \left( {p_{i}^{4\prime } p_{i}^{3\prime } p_{i + 1}^{3\prime } p_{i}^{2\prime } p_{i + 1}^{2\prime } p_{i}^{1\prime } p_{i + 1}^{1\prime } } \right)$$, $$\left( {d_{i} d_{i + 1} d_{i + 2} } \right) = (b_{1}^{\prime } b_{2}^{\prime } \ldots b_{7}^{\prime } ) \times {\varvec{H}}^{\text{T}}$$, $$i \in \{ N_{1} + 1, N_{1} + 3, N_{1} + 5, \ldots , N_{1} + 2N_{2} - 1\}$$.

**Step 4:**$$(b_{1}^{\prime } b_{2}^{\prime } \ldots b_{7}^{\prime } ) = \left( {p_{i}^{3\prime } p_{i}^{2\prime } p_{i + 1}^{2\prime } p_{i + 2}^{2\prime } p_{i}^{1\prime } p_{i + 1}^{1\prime } p_{i + 2}^{1\prime } } \right)$$, $$\left( {d_{i} d_{i + 1} d_{i + 2} } \right) = (b_{1}^{\prime } b_{2}^{\prime } \ldots b_{7}^{\prime } ) \times {\varvec{H}}^{\text{T}}$$, $$i \in \{ N_{1} + 2N_{2} + 1, N_{1} + 2N_{2} + 4, \ldots , N_{1} + 2N_{2} + 3N_{3} - 2\}$$.

### Example: data extracting

Suppose the receiver receives the marked-image sized *H* × *W* = 3 × 3 shown in Fig. 2 and knows that the embedding rate (ER) is 2 bpp.

**Step 1:** Work out *N*_1_ = 3, *N*_2_ = 3, *N*_3_ = 0 by Formula (5).

**Step 2:**$p_{ 1}^{'} = 163,(b_{1}^{\prime } b_{2}^{\prime } \ldots b_{7}^{\prime } ) = (p_{1}^{7\prime } p_{1}^{6\prime } \ldots p_{1}^{1\prime } ) = \left( {0100010} \right)$, $\left( {d_{1} d_{2} d_{3} } \right) = (b_{1}^{\prime } b_{2}^{\prime } \ldots b_{7}^{\prime } ) \times {\varvec{H}}^{\text{T}} = \left( {101} \right)$. Repeat Step 2 (*N*_1_ − 1) times to extract the bits embedded in $p_{2}^{\prime } = 163,p_{3}^{\prime } = 158$.

**Step 3:**$\left( {p_{4}^{\prime } , p_{5}^{\prime } } \right) = \left( {162,151} \right)$, $(b_{1}^{\prime } b_{2}^{\prime } \ldots b_{7}^{\prime } ) = \left( {p_{3}^{4\prime } p_{3}^{3\prime } p_{4}^{3\prime } p_{3}^{2\prime } p_{4}^{2\prime } p_{3}^{1\prime } p_{4}^{1\prime } } \right) = \left( {0001001} \right)$, $\left( {d_{10} d_{11} d_{12} } \right) = (b_{1}^{'} b_{2}^{'} \ldots b_{7}^{'} ) \times {\varvec{H}}^{\text{T}} = \left( {010} \right)$. Repeat Step 3 (*N*_2_ − 1) times to extract the bits embedded in $\left( {p_{6}^{\prime } , p_{7}^{\prime } } \right) = \left( {154, \, 165} \right), \, \left( {p_{8}^{\prime } , p_{9}^{\prime } } \right) = \left( {151, \, 152} \right)$.

## Experiment results

**Fig. 3:**
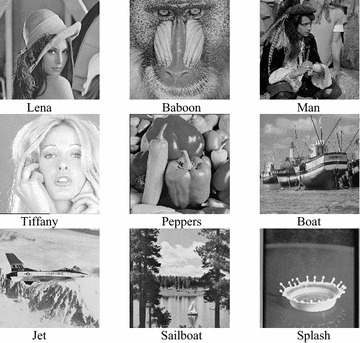
The nine test images

To evaluate the performance of the proposed scheme, we simulate the “Matrix Encoding” (Crandall 1998), the “Hamming+1” (Zhang et al. 2007), the “Nearest Code” (Chang and Chou 2008) and the proposed scheme by software Matlab R2014a. Standard grayscale test images sized 512 × 512 are used in the simulations, as shown in Fig. 3.

### Preprocessing

In order to make comparison objectively and fairly, the embedding capacity of “Matrix Encoding” (Crandall 1998), “Hamming+1” (Zhang et al. 2007) and “Nearest Code” (Chang and Chou 2008) are also enhanced by extending the least significant bit to general LSBs. The extension method of “Matrix Encoding” is as follows. Every 3-bit string is embedded into ${\varvec{G}}_{i} (1 \le i \le 7)$ which is composed of the *i*-th least significant bit of 7 pixels by the matrix encoding method. Thus, the embedding capacity of the extended “Matrix Encoding” method become 3 bpp.

The “Hamming+1” scheme is extended as follows. Every 4-bit string is embedded into ${\varvec{G}}_{i} (1 \le i \le 4)$, composed of the (2*i* − 1)-th and 2*i*-th least significant bits of 8 pixels, using the “Hamming+1” method. Thus, the embedding capacity of the extended “Hamming+1” scheme become 2 bpp.

Also, the extension method of the “Nearest Code” is as follows. Every 3-bit string is embedded into ${\varvec{G}}_{i} (1 \le i \le 7)$ which is composed of the *i*-th least significant bit of 7 pixels by the “Nearest Code” method, making the embedding capacity of the extended “Nearest Code” method be 3 bpp.

To be fair to compare with the related works, the same method used in obtaining Formula (5) is applied here to process the extended “Matrix Encoding”, “Hamming+1” and “Nearest Code” to be adaptive to the payload as follows.

The extended “Matrix Encoding”:

6 $$\left\{ {\begin{array}{ll} {N_{1} = L} &\quad {ER \le \frac{3}{7}} \\ {N_{i} = \frac{i + 1}{7}H \times W - L,N_{i + 1} = L - \frac{i}{7}H \times W} &\quad {\frac{3i}{7} < ER \le \frac{3(i + 1)}{7}(1 \le i \le 6)} \\ \end{array} } \right.$$

The extended “Hamming+1”:

7 $$\left\{ {\begin{array}{ll} {N_{1} = L} & \quad {ER \le \frac{1}{2}} \\ {N_{i} = \frac{i + 1}{8}H \times W - L,N_{i + 1} = L - \frac{i}{8}H \times W} & \quad {\frac{i}{2} < ER \le \frac{i + 1}{2}(1 \le i \le 3)} \\ \end{array} } \right.$$

The extended “Nearest Code”:

8 $$\left\{ {\begin{array}{ll} {N_{1} = L} &\quad {ER \le \frac{3}{7}} \\ {N_{i} = \frac{i + 1}{7}H \times W - L,N_{i + 1} = L - \frac{i}{7}H \times W} &\quad {\frac{3i}{7} < ER \le \frac{3(i + 1)}{7}( 1\le i \le 6)} \\ \end{array} } \right.$$

where, *N*_*i*_ represents the groups of data bits embedded in ${\varvec{G}}_{i}$.

### Image quality

PSNR (Peak Signal to Noise Ratio) is widely used to measure the image quality of marked-images by calculate the difference between the marked-image and the cover image, which is defined as follows.

9 $${\text{PSNR}} = 1 0 {\text{log}}_{ 1 0} \frac{{ 2 5 5^{ 2} }}{\text{MSE}} ( {\text{dB)}}$$

10 $${\text{MSE}} = \frac{ 1}{H \times W}\sum\limits_{i = 1}^{H} {\sum\limits_{j = 1}^{W} {(I_{i,j} } - I_{i,j}^{{\prime }} )^{2} }$$

The above equations demonstrate that the smaller the difference between the marked-image and cover image is, the greater the PSNR value is. In general, if a marked-image with PSNR value greater than 30 dB, the distortion of the marked-image is hard to be detected by human eyes.

Tables 1, 2, 3 and 4 show the PSNR values of marked images generated by different methods with several payloads, i.e. ER = 1 bpp, ER = 1.5 bpp, ER = 2 bpp and ER = 3 bpp. The data in tables are the mean value of ten independent experiments. And data bits embedded into images are generated randomly. From the tables, it’s obvious that the PSNR values of the proposed scheme are higher than those of the related works. It indicates that the marked-image quality of the proposed scheme is superior to those of the related works under the same payload.

**Table 1:** The PSNR comparison of different methods with ER = 1 bpp

	Lena	Baboon	Man	Tiffany	Peppers	Boat	Jet	Sailboat	Splash
Matrix encoding	47.02	47.02	47.03	47.02	47.02	47.01	47.04	47.01	47.03
Nearest code	47.02	47.02	47.01	47.03	47.02	47.01	47.04	47.01	47.03
Hamming+1	45.14	45.14	45.01	45.10	45.14	45.14	45.14	45.14	45.13
Proposed scheme	51.14	51.14	51.14	51.15	51.14	51.14	51.15	51.14	51.14

**Table 2:** The PSNR comparison of different methods with ER = 1.5 bpp

	Lena	Baboon	Man	Tiffany	Peppers	Boat	Jet	Sailboat	Splash
Matrix encoding	39.90	39.92	39.92	39.93	39.92	39.94	39.93	39.93	39.88
Nearest code	39.90	39.91	39.92	39.93	39.91	39.94	39.93	39.93	39.88
Hamming+1	33.08	33.10	32.73	32.77	33.04	33.05	33.08	33.09	33.01
Proposed scheme	46.37	46.37	46.37	46.38	46.37	46.36	46.38	46.37	46.37

**Table 3:** The PSNR comparison of different methods with ER = 2 bpp

	Lena	Baboon	Man	Tiffany	Peppers	Boat	Jet	Sailboat	Splash
Matrix encoding	33.10	33.08	33.06	33.09	33.05	33.10	33.10	33.06	33.18
Nearest code	33.11	33.07	33.06	33.09	33.06	33.10	33.10	33.07	33.18
Hamming+1	20.62	20.85	20.25	19.78	20.63	20.70	19.98	20.27	20.54
Proposed scheme	41.61	41.60	41.62	41.57	41.60	41.58	41.67	41.59	41.62

**Table 4:** The PSNR comparison of different methods with ER = 3 bpp

	Lena	Baboon	Man	Tiffany	Peppers	Boat	Jet	Sailboat	Splash
Matrix encoding	19.80	19.77	19.63	19.87	19.89	19.70	20.07	20.09	19.82
Nearest code	19.80	19.77	19.64	19.87	19.89	19.69	20.08	20.09	19.81
Hamming+1	–	–	–	–	–	–	–	–	–
Proposed scheme	37.92	37.92	37.92	37.91	37.92	37.92	37.98	37.89	37.94

**Fig. 4:**
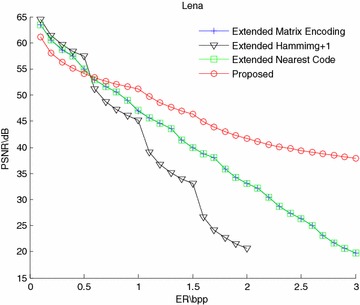
PSNR-ER comparison of Lena

**Fig. 5:**
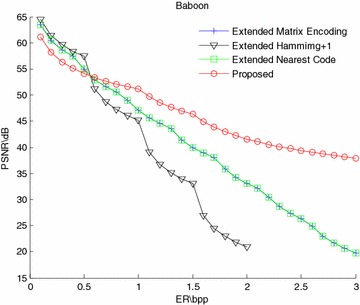
PSNR-ER comparison of Baboon

The PSNR-ER comparison results of Lena and Baboon are shown in Figs. 4 and 5. From the figures, the PSNR values of the proposed scheme are slightly lower than those of the extended “Matrix Encoding”, “Hamming+1” and “Nearest Code” schemes when the embedding rate is relatively small, but while the embedding rate gets greater, the PSNR values of the proposed scheme are significantly higher than those of the other methods. By the way, the curves of the “Extend Matrix Encoding” and the “Extend Nearest Code” are completely overlapped, because both of the two methods embed three bits by modifying one bit. Thus, only the results of the extended “Matrix Encoding” scheme are shown in the next experiment results.

**Fig. 6:**
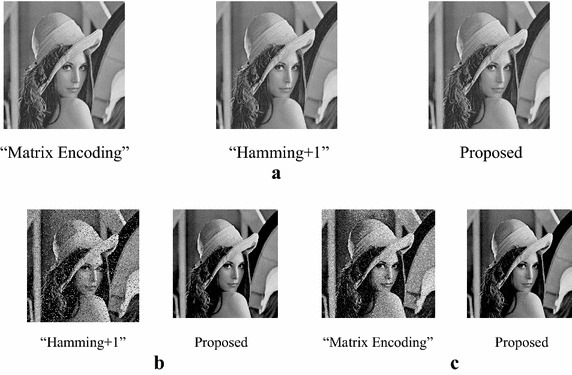
Marked-images of Lena under various payloads. **a** ER = 3/7 bpp. **b** ER = 2 bpp. **c** ER = 3 bpp

**Fig. 7:**
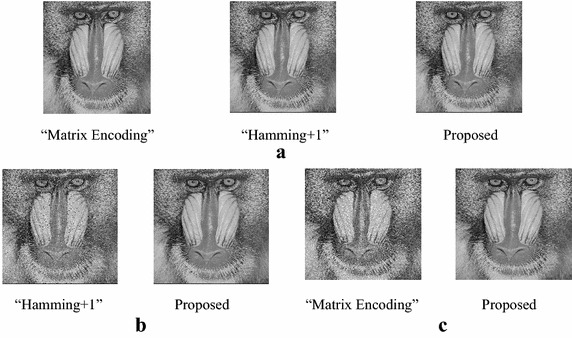
Marked-images of Baboon under various payloads. **a** ER = 3/7 bpp. **b** ER = 2 bpp. **c** ER = 3 bpp

Take Lena and Baboon for examples, the marked-images of the extended “Matrix Encoding”, the extended “Hamming+1”and the proposed scheme under different payloads are shown in Figs. 6 and 7. We can see that there is no distinct difference between the marked-images when the embedding rate is 3/7 bpp. When the embedding rate is up to 2 bpp, we can see spots easily on the marked-image of the extended “Hamming+1” scheme, and can hardly see any spot on the marked-image of the proposed scheme. The same observations can be found between the proposed scheme and the extended “Matrix Encoding” scheme while ER = 3 bpp.

### Security analysis

**Fig. 8:**
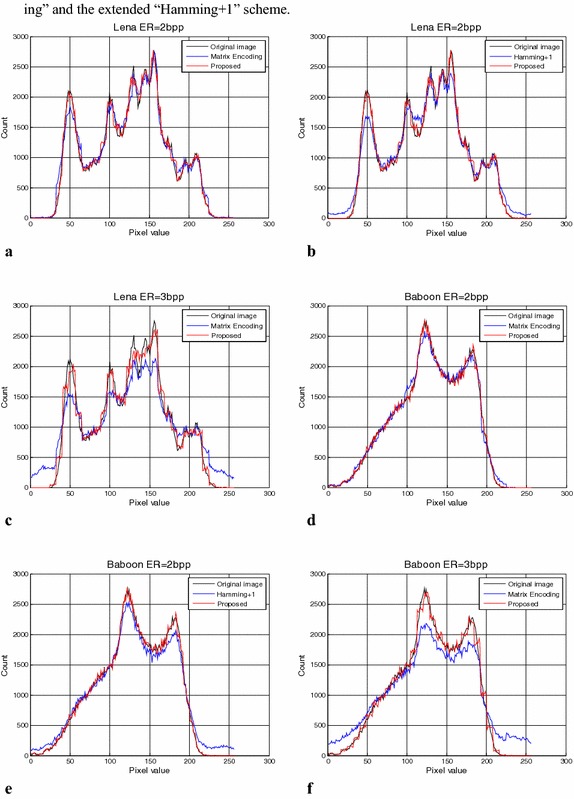
The pixel histogram analysis comparison of Lena and Baboon. **a** “Matrix Encoding” of Lena with ER = 2 bpp. **b** “Hamming+1” of Lena with ER = 2 bpp. **c** “Matrix Encoding” of Lena with ER = 3 bpp. **d** “Matrix Encoding” of Baboon with ER = 2. **e** “Hamming+1” of Baboon with ER = 2 bpp. **f** “Matrix Encoding” of Baboon with ER = 3

Security is a significant problem for data hiding. Many steganalysis methods uses statistics tools to analysis the pixel value distribution on a suspicious image for cracking the secret message delivery. From this point of view, we analysis the pixel histograms between the test cover images and the marked images to measure the security of the data hiding methods. Take a smooth content image Lena and a complex content image Baboon for example, the pixel histogram results generated by the extended “Matrix Encoding” scheme, the extended “Hamming+1” scheme, and the proposed scheme with high payloads are shown in Fig. 8. From Fig. 8, the pixel histogram of the marked image generated by the proposed scheme is closer to the pixel histogram of the original image than those of the extended “Matrix Encoding” and extended “Hamming+1” scheme. It demonstrate that the security performance of the proposed scheme is better than the extended “Matrix Encoding” and the extended “Hamming+1” scheme.

## Conclusions

Based on (7, 4) Hamming code, a novel high capacity data hiding scheme is proposed. Cover pixels are matched adaptively to embed data according to different embedding payloads. Compared to the related works, the image quality under high payload gets improved significantly while maintaining visual quality under low payload. Because of the use of pixel matching, a seed can be also used to match pixels to improve the security. Moreover, this method is not limited to grayscale images, but can be also applied to color images, compressed images, audios, videos and other digital media. Future works include investigating this scheme on other error correcting codes and improving the data embedding efficiency further.

## References

[CR1] Chang C, Chou Y (2008) Using nearest covering codes to embed secret information in gray scale images. In: Proceedings of the 2nd international conference on ubiquitous information management and communication. ACM, pp 315–320

[CR2] Chen L, Lu L, Hu A, Sun X (2013). Improved information hiding algorithm based on twice positioning in coding channel. J Commun.

[CR3] Crandall R (1998) Some notes on steganography. Posted on steganography mailing list. http://os.inf.tudresden.de/westfeld/Crandall.pdf

[CR4] Feng G, Lan Y, Zhang X, Qian Z (2015). Dynamic adjustment of hidden node parameters for extreme learning machine. IEEE Trans Cybern.

[CR5] Hong W, Chen T, Chen J (2015). Reversible data hiding using Delaunay triangulation and selective embedment. Inf Sci.

[CR6] Ker A, Bas P, Böhme R, Cogranne R, Craver S, Filler T, Fridrich J, Pevny T (2013) Moving steganography and steganalysis from the laboratory into the real world. In: Proceedings of the First ACM workshop on information hiding and multimedia security. ACM, pp 45–58

[CR7] Liu C (2007) Research on theory and application of steganography based on error-correcting code. Dissertation, the PLA Information Engineering University

[CR8] Ma Z, Li F, Zhang X (2013). Data hiding in halftone images based on hamming code and slave pixels. J Shanghai Univ (Nat Sci).

[CR9] Qian Z, Zhang X (2015). Reversible data hiding in encrypted image with distributed source encoding. IEEE Trans Circuits Syst Video.

[CR10] Wang X (2009) Research on channel coding based information hiding techniques. Dissertation, Harbin Institute of Technology

[CR11] Xia Z, Wang X, Sun X, Wang B (2014). Steganalysis of least significant bit matching using multi-order differences. Secur Commun Netw.

[CR12] Xia Z, Wang X, Sun X, Liu Q, Xiong N (2014) Steganalysis of LSB matching using differences between nonadjacent pixels. Multimed Tools Appl 1–16. doi:10.1007/s11042-014-2381-8

[CR13] Yin Z, Chang C, Zhang Y (2010). An information hiding scheme based on (7, 4) hamming code oriented wet paper codes. IJICIC.

[CR14] Zhang W, Wang S, Zhang X (2007). Improving embedding efficiency of covering codes for applications in steganography. IEEE Commun Lett.

[CR15] Zhu X, Liu J, Zhang W (2010). A steganographic algorithm based on hamming code and wet paper code. J Electron Inf Technol.

[CR16] Zielińska E, Mazurczyk W, Szczypiorski K (2014). Trends in steganography. Commun ACM.

